# A Call for Integrated Psychiatry-Psychology Collaboration on Consult-Liaison Services: Experiences of a Psychology Extern and Recommendations for Collaborative Care

**DOI:** 10.7759/cureus.43874

**Published:** 2023-08-21

**Authors:** Audrey D Nguyen, Osmara Medrano, Saba Syed

**Affiliations:** 1 Psychiatry, University of California Los Angeles, David Geffen School of Medicine, Los Angeles, USA; 2 Psychiatry, Olive View-University of California Los Angeles (UCLA) Medical Center, Los Angeles, USA

**Keywords:** consult-liaison psychiatry, psychological interventions, integrated care, collaborative care, serious mental illness

## Abstract

Few institutions have integrated psychologists into consult-liaison (CL) psychiatry services caring for patients with medical conditions and comorbid serious mental illnesses (SMI). To our knowledge, no studies have explored the experiences of an integrated pre-doctoral psychologist on a CL psychiatry service and applications of collaborative care. The present study aims to 1) highlight the experiences of a psychology extern on an adult CL psychiatry service in a public academic hospital, and 2) apply a collaborative care framework to provide recommendations for implementing integrated psychiatry-psychology collaboration. A semi-structured qualitative interview was performed with the team’s psychology extern to elicit practice goals and setting. As of June 2022, the psychology extern saw 76 inpatient adults over the year-long externship period. Through diverse case vignettes, we illustrate the extern’s unique roles in providing psychotherapeutic interventions to enhance patients’ coping skills. We further found that embedding a psychology extern within the CL psychiatry service follows the integrated model of collaborative care. We thus apply a collaborative care framework to guide CL services in using multidisciplinary teams to improve care quality for inpatient adults. Leveraging the expertise of a psychology extern in real-time collaboration with CL psychiatry teams can enhance patient-centered care and warrants broader institutional implementation. Future studies are needed to explore the efficacy of integrated psychiatry-psychology collaboration on provider perspectives and clinical outcomes.

## Introduction

Among patients with serious mental illness (SMI) (e.g., psychotic disorders or major mood disorders), morbidity and mortality risks are elevated. Of the excess deaths among patients with SMI, 30-40% are attributable to suicide and injuries, and 60-70% are due to comorbid medical conditions [1]. Behavioral factors such as substance use [2] and difficulties with medication adherence [3] may exacerbate underlying conditions. In fact, maladaptive coping behaviors, defined as avoidance-oriented (e.g., denying the problem) or emotion-oriented (e.g., self-criticism) are more common among patients with SMI compared to healthy individuals [4,5]. Maladaptive coping has been associated with reduced quality of life and chronic disease progression [6]. In contrast, adaptive coping behaviors have been associated with reduced severity of mental illness [7] and enhanced quality of life [8]. Improving coping behaviors for patients with SMI therefore has potential to improve outcomes, and collaborative efforts between psychiatrists and psychologists can be critical in addressing this need.

In consult-liaison (CL) psychiatry, hospitalized patients with medical (physical) conditions and comorbid psychiatric disorders are cared for by an interdisciplinary team. While psychologists may be trained as part of CL teams, few institutions have adopted this practice [9,10]. In fact, there is no reliable published data available that shows the proportion of CL psychiatry services in the United States with integrated psychologists and their level of training. Additionally, previous studies have examined the implications of adding a CL rotation for psychology internship training. They found that CL psychology interns greatly expanded the scope of services offered [11] and were able to acquire novel skills such as decision-making capacity evaluations [12]. These studies focused on the development and impact of CL rotations in training psychologists. Rather than internship-level psychology training, Rutledge et al. summarized an interprofessional psychology fellowship program in CL psychiatry and suggested the need for more experience-centered examination of CL psychologists [13].

The experiences of psychologists in CL psychiatry remain underexplored. To our knowledge, no studies have investigated integrated psychology collaboration on CL psychiatry teams and examined their experiences through a collaborative care framework. We thus highlight a psychology externship program to call for broader implementation of integrated psychiatry-psychology collaboration on CL psychiatry services. The primary aims are to 1) highlight the roles and experiences of a psychology extern in caring for patients on an adult CL psychiatry service in a public academic hospital, and 2) apply a collaborative care framework to provide recommendations for implementing integrated psychology collaboration on adult CL psychiatry services. Leveraging expertise from psychologists in real-time collaboration with psychiatrists has potential to improve patient-centered care and coping skills for hospitalized adults with comorbid SMI and medical conditions.

Of note, this article was previously presented as a poster at the 2023 American Psychiatric Association Annual Meeting on May 21, 2023.

## Materials and methods

Study design

First, we conducted a semi-structured qualitative interview with the CL psychiatry team’s psychology extern to explore the practice setting, goals for contribution, patient presentations, and psychological interventions implemented. Next, we examined the role of collaborative care to develop recommendations for the implementation of integrated care between psychologists and psychiatrists in CL psychiatry.

Description of psychology externship

The psychology externship program consists of one psychology extern, typically a pre-doctoral, pre-internship psychology student, who is present on the CL psychiatry team for 12 months and supervised by the attending psychiatrists. The extern’s specific goals included 1) exploring the impact of psychological factors and co-morbid psychiatric disorders on the course of medical illnesses, with particular attention to maladjustment to illness, 2) helping patients cope with illness and/or hospitalization utilizing psychological interventions, and 3) working collaboratively with interdisciplinary providers including psychiatry residents, social workers, and specialty physicians. The overarching goal is to integrate biological, psychological, and socio-cultural domains into treatment to promote better management of distressing emotions, improve adaptive behaviors, and enhance social self-care and connections to resources.

The role of the psychology extern involves participation in in-person daily rounds to discuss patients’ medical and psychological problems with the medical team, which consists of medical students, residents, and two attending psychiatrists. Patients are subsequently assigned to the extern that are likely to benefit from one-to-one psychoeducation and therapy sessions during their admission. The patients assigned to the psychology extern are selected based on their openness to therapy and their ability to engage in sessions according to their cognitive capacity and psychological functioning. Finally, the extern documents clinical interventions in the electronic medical record and assists in discharge planning. Supervision of the extern is provided by attending psychiatrists on the CL service and oversight is provided by the director of the psychology training program.

Once the extern is assigned to patients, she utilizes a single-session therapy (SST) framework to promote active patient engagement in managing their medical and psychiatric conditions, substance use, and psychosocial stressors that contributed to hospitalization [14]. The application of single-session interventions (SSI) has the potential to maximize growth in settings in which multiple sessions may not be available [14]. SSIs draw from evidence-based components that allow the extern to curate individualized sessions that emphasize abilities and resources, rather than pathology [14].

Sessions last between 20-59 minutes and utilize a myriad of techniques to support the patient. First, the extern provides psychoeducation to the patient and presents the possibility for additional inpatient sessions, as the inpatient setting is frequently focused on medical stability discharge criteria that may result in a short duration of stay and lack of therapy continuity [15]. Several crucial concepts are identified during this portion of the session, such as understanding the patient’s sense of autonomy in an acute setting [15]. Motivational interviewing (MI) techniques are employed to better evaluate the patient’s motivation state of readiness to change [16,17].

Then, the extern acquires patient history, including preceding triggers or events that led to the current hospitalization, acute or chronic psychosocial stressors, and the patient’s mechanisms for coping with these stressors. Next, she performs a thorough mental status exam, particularly noting nonverbal behavioral observations (e.g., pacing in the room, dyskinesia-like symptoms) and suicidal or homicidal ideation. Finally, either during the SST or in a subsequent session, the extern provides psychological interventions to address the unique needs of the patient.

Psychological interventions

Previous research has demonstrated the impact of delivering psychological interventions in an acute setting. For instance, psychological interventions have been associated with reduced hospital readmissions, anxiety, and depression [18]. They have also been associated with improved post-intervention symptoms such as treatment compliance, decreased risk of re-hospitalization, and social functioning [19]. The extern selects psychological interventions based on several factors, including clinical history obtained from chart review of Department of Mental Health and Department of Health Services documentation, the initial patient interview, treatment planning during rounds, and the patient’s self-reported evaluation acquired via validated scales such as the Patient Health Questionnaire-9 or Generalized Anxiety Questionnaire. Depending on their prior level of experience in psychotherapy, the psychology extern either independently selects the psychological interventions with guidance by the attending psychiatrist or collaboratively selects the interventions.

In particular, the extern utilizes an integrative therapeutic approach that includes evidence-based practices such as cognitive behavioral therapy (CBT), dialectical behavioral therapy (DBT), and motivational interviewing (MI) (Table 1). In brief, CBT techniques helped direct patients toward solving and modifying current problem behaviors [20]. DBT interventions helped promote how to change behaviors and improve flexibility during the change process [21]. Lastly, MI interventions assisted patients in identifying and changing behavior and potential resistance that contributes to ongoing chronic conditions [16].

**Table 1:** Description of Psychological Interventions and Techniques Various evidence-based therapies, including cognitive behavioral therapy, dialectic behavioral therapy, and motivational interviewing, are used by the psychology extern on the CL psychiatry service. These interventions are delivered either alone or in combination during single-session therapy sessions and tailored according to patient needs and motivations.

Cognitive Behavioral Therapy (CBT) Interventions		
Intervention	Definition	Example
Automatic Thoughts (AT) [20]	AT are thoughts that influence one’s emotion, behaviors, and physiological response.	Help identify, respond, and evaluate AT that are negative or inaccurate.
Cognitive Restructuring [20]	Assessing and responding to maladaptive thinking.	Utilize questioning to help uncover irrational or unhelpful thinking.
Adaptive Coping Strategies [20]	This can include mindfulness, imagery, relaxation exercises.	Identify unique ways to regulate and tolerate distressing situations.
Imagery [20]	Images can be sensory or somatic in addition to visual and affect how we feel.	Promote positive imagery to focus on coping or solving situations.
Dialectic Behavioral Therapy (DBT) Interventions		
Intervention	Definition	Example
Core Mindfulness [21]	Intentionally paying attention to the moment, without judging it or holding on to it.	Utilizing present interactions to help focus attention on sensations and thoughts and letting go.
Dialectics [21]	The dialectic between the need for clients to accept themselves as they are in the moment and the need for them to change.	Provide psychoeducation about the tension between acceptance and change.
Emotional Regulation [21]	The ability to change individual responses (impulsive and inappropriate behavior), and self-soothe.	Help identify intense and triggering emotions that lead to somatic symptoms.
Distress Tolerance [21]	The ability to tolerate and accept distress.	Explore ways patients have coped and self-soothe with past situations and teach distress skills.
Interpersonal Effectiveness [21]	Focuses on decreasing pain and suffering by effectively interacting with social environment.	Help become more effective in interactions with others (communication, expressing).
Chain “behavior” Analysis [21]	A chain analysis examines the chain of events that leads to ineffective behaviors, as well as consequences.	Begin with problem behavior that involves vulnerabilities and ways to create reparation.
Motivational Interviewing (MI) Interventions		
Intervention	Definition	Example
OARS [17]	Open-ended questions, Affirmations, Reflective listening, Summarizing.	Help facilitate transparent discussion about problem areas while promoting positive traits.
Rolling with Resistance [16]	The aim is to not argue with the statement but delicately challenge the thought process that underlies the behavior one wants to change.	Allow for resistance to exist without judgment and identify the conflict between problems and solutions.
Developing Discrepancy [16]	Discrepancy is amplified between where the patient is currently and where they want to be.	Help develop goals and grow awareness of the space between present and future goals.
Removing Barriers [16]	Explore barriers to receiving particular access to particular help.	Engage the patient in observing past barriers that “get in the way” of improvement.

## Results

Patient characteristics

The CL service receives approximately nine to 12 consult requests per week. The psychology extern will typically be assigned to follow approximately 30-40% of those patients. As of June 2022, the extern has seen 76 inpatient adults over the year-long externship period. Of the 76 patients seen, the majority were male adults between ages 18-64 years and primarily of Latino-a/Hispanic ethnicity (Table 2). The patients seen had been placed on an involuntary psychiatric hold, typically either a 72-hour or extended psychiatric hold (14-day) due to endorsed suicidality or suicidal attempt, or were considered gravely disabled.

**Table 2:** Demographic Summary of Adult Inpatients Seen by Psychology Extern

Sample Characteristics	N = 76	%
Gender		
Men	49	65
Women	26	34
Transgender	1	1
Age		
18-64 years old	62	81.6
65 years old or older	14	18.4
Race		
African American/Black	4	5.3
Asian-American/Asian Origin/Pacific Islander	3	3.9
Latino-a/Hispanic	40	52.6
European Origin/White	24	31.6
Bi-racial/Multi-racial	1	1.3
Other/Unidentified	4	5.3
Sexual Orientation		
Heterosexual	67	88.2
Gay	6	7.9
Unidentified	3	3.9

Therapy sessions

Most of the psychological encounters were conducted in person, with 97% (74/76) occurring in the patient’s hospital room and 3% (2/76) being adaptations in which the extern interviewed the patient via telephone from the window outside the room due to COVID-19 positive status. Session flexibility regarding time and length is necessary for an acute inpatient setting; the patients were thus seen on a weekday between Monday and Friday in the mornings. The median duration for brief therapeutic sessions was estimated to be 40 minutes. Among the encounters, 43/76 (56.5%) lasted 40-59 minutes, while 33/76 (43%) lasted 20-39 minutes.

Clinical case vignettes

We selected three patient case examples to illustrate unique aspects and outcomes of providing patient-tailored, brief psychotherapy in an inpatient setting.

Case #1

An elderly patient with a history of schizophrenia was brought to the hospital by a homeless shelter due to a grave disability and was found to have stomach cancer. The CL service was consulted for treatment recommendations for schizophrenia.

Interventions chosen: The patient presented with challenges in communication and medication adherence. Psychosocial factors contributing to his presentation included limited social support and housing instability. Psychological interventions were chosen to foster better communication and social/interpersonal skills to enhance engagement with the treatment team. The extern cultivated trust with the patient through open dialogue about his experience in the hospital, and the patient ultimately displayed reduced guardedness toward her. The extern also modeled interpersonal behaviors and reinforced positive social behaviors during sessions. Additionally, social interaction with the extern allowed for behavior rehearsal and led to better communication with hospital staff. Following three sessions, the patient demonstrated improved behaviors during his hospital stay through increased verbal communication of needs, calmer interactions with other hospital staff, and improved medication adherence.

Case #2

A middle-aged male with major depressive disorder with psychosis, post-traumatic stress disorder (PTSD), and active COVID-19 infection was brought in for alcohol withdrawal symptoms and suicidal ideation. CL psychiatry was consulted due to a suicide attempt. Due to potential COVID-19 exposure, the extern interviewed the patient via telephone while simultaneously visualizing him through a window.

Interventions chosen: The patient presented with depressive symptoms and difficulty with challenging negative thoughts. Psychosocial factors included limited social support, unstable housing, and polysubstance use. The interventions chosen included CBT combined with MI techniques to foster alternative options and changing behavior. The extern met with the patient for two sessions and established visual contact through the hospital room window due to the patient’s COVID-19 status. The patient demonstrated engagement by sitting up at the edge of the bed and nodding that he was willing to engage in the session. The extern reviewed previously discussed depressive symptoms and CBT techniques, such as challenging negative automatic thoughts, examining the thoughts, and how they influence maladaptive behavior. After the session, the patient exhibited improved ability to recognize alternative thoughts and reduce negative thoughts about himself and others that influenced maladaptive coping behaviors. The patient also showed an improved ability to practice homework related to managing negative thoughts about self and others, as well as decreased suicidality. Finally, the patient expressed openness to outpatient mental health treatment.

Case #3

A young female with a history of major depressive disorder and previous suicidal attempts was admitted for poor oral intake, behavioral dysregulation, and suicidal ideation.

Interventions chosen: The patient presented with difficulty with emotional regulation and persistent suicidal ideation. Psychosocial factors included relational conflict with a romantic partner. Chosen interventions included supportive therapy and DBT interventions to support the patient’s ability to gain insight about the vulnerabilities that contributed to her hospitalization. The extern met with the patient for two sessions, which included rapport building, identifying target behaviors, and examining cognitive-behavioral components that led to life-threatening behaviors. Once therapy-interfering behaviors such as relationships and non-compliance were recognized, the session was focused on decreasing negative behaviors while promoting social and effective behavioral skills, including emotion regulation, distress tolerance, and completion of a chain analysis (Table 1). Particularly with highly impulsive behaviors, the chain analysis facilitated more insight into her behaviors and vulnerabilities that led to the problem behavior. Following interventions, the patient expressed reduced passive suicidal thoughts, improved oral intake, and more cooperation in her interactions with hospital staff.

## Discussion

Summary of findings

The present study describes the experiences of a psychology extern on the adult CL psychiatry service at a single academic site. The extern cares for patients with diverse demographic characteristics reflective of the population served by the hospital. Through case vignettes, we further showed applications of individualized therapeutic interventions for adult inpatients with various clinical presentations and their effects on patients’ coping behaviors and participation in care. By examining the psychiatry-psychology relationship through a collaborative care framework, we further highlight the potential for the broader application of integrated psychiatry-psychology collaboration in CL psychiatry services.

Role of psychologists in CL services

Many patients with SMI on the CL service have multiple medical, substance, and psychiatric comorbidities, requiring multidisciplinary teams with transparent, collaborative approaches. CL services can therefore be a cornerstone for changing patient-centered healthcare delivery by integrating psychologists across hospital settings. The role of integrated psychologists in inpatient CL services has been underexplored. However, psychologists have been noted as beneficial contributors in other services. In palliative care, for example, they develop psychotherapeutic relationships, conduct psychological assessments, and provide in-depth knowledge about end-of-life care [22]. In primary care, child psychologists and child psychiatrists screen patients for psychosocial stressors and psychopharmacologic issues (e.g., safe medication usage) [23]. Similar to other medical specialties, the psychology extern in this study was valuable in developing patient relationships, conducting psychological screenings, and providing recommendations for coping interventions that complemented the medication management and better engaged patients in care participation.

Applying collaborative care in CL services

When the role of a psychology extern has been established on CL services, the nature of collaboration between psychiatry and psychology should follow a collaborative care model. The Substance Abuse and Mental Health Services Administration describes three categories of collaborative care that can be applied in mental health: Coordinated, Co-located, and Integrated Care, with levels 1-6 corresponding to the increasing extent of collaboration between primary teams and mental health providers [24]. A summary of key points describing each model is shown in Table 3.

**Table 3:** Core Descriptions of the Levels of Collaborative Care Between Behavioral Health and Medical Providers Increasing levels (1-6) represent increasing levels of collaboration. The psychiatry-psychology collaboration discussed in the present study contains pertinent aspects of the Integrated model described below, primarily encompassing levels 5 and 6. Table adapted with permission from the SAMHSA-HRSA Center for Integrated Health Solutions [24]. ^a^Components of the Integrated model that are utilized by the psychiatry-psychology collaboration discussed in the present study

COORDINATED		CO-LOCATED		INTEGRATED	
Key Element: Communication		Key Element: Physical Proximity		Key Element: Practice Change	
Level 1: Minimal Collaboration	Level 2: Basic collaboration at a distance	Level 3: Basic Collaboration onsite	Level 4: Close collaboration onsite with some system integration	Level 5: Close collaboration approaching an integrated practice	Level 6: Full collaboration in a transformed/merged integrated practice
Behavioral health, primary care and other healthcare providers work:					
In separate facilities, where they:	In separate facilities, where they:	In the same facility not necessarily the same offices, where they:	In the same space within the same facility, where they:	In the same space within the same facility (some shared space), where they:	In the same space within the same facility, sharing all practice space, where they:
Have separate systems; Communicate about cases only rarely and under compelling circumstances; Communicate, driven by provider need; May never meet in person; Have limited understanding of each other’s roles.	Have separate systems; Communicate periodically about shared patients; Communicate, driven by specific patient issues; May meet as part of a larger community; Appreciate each other’s roles as resources.	Have separate systems; Communicate regularly about shared patients, by phone or e-mail; Collaborate, driven by the need for each other’s services and more reliable referral; Meet occasionally to discuss cases due to close proximity; Feel part of a larger yet non-formal team.	Share some systems, like scheduling or medical records; Communicate in person as needed; Collaborate, driven by the need for consultation and coordinated plans for difficult patients; Have regular face-to-face interactions about some patients; Have a basic understanding of roles and culture.	Actively seek system solutions together or develop work-a-rounds^a^; Communicate frequently in person^a^; Collaborate, driven by the desire to be a member of the care team^a^; Have regular team meetings to discuss overall patient care and specific patient issues^a^; Have an in-depth understanding of roles and culture^a^	Have resolved most or all system issues, functioning as one integrated system; Communicate consistently at the system, team, and individual levels^a^; Collaborate, driven by a shared concept of team care^a^; Have formal and informal meetings to support integrated model of care^a^; Have roles and cultures that blur or blend.

In the present study, embedding a psychology extern within the CL team aligns closely with the integrated model of collaborative care (Table 3). In this CL service, the extern shared the same physical spaces as psychiatry residents and engaged in frequent and almost daily in-person interactions, allowing for the development of collective goals driven by a shared commitment to solving patient issues. These conditions enabled the initiation and maintenance of a sustained collaboration. While the psychiatrists coordinated medication management, the psychology extern provided inpatient psychotherapeutic support that targeted patients’ coping skills, allowing for patients to improve medication adherence, suicidal ideation, and/or engagement in care. Similarly, with the psychology extern participating in daily treatment rounds, the extern remained aware of patients’ medical issues as managed by the primary team, allowing for a comprehensive understanding of patient care goals.

In addition, collaborative care has primarily been studied in outpatient settings, where the collaborative care team generally consists of a primary provider, psychiatrists, and behavioral health providers (e.g., psychiatric nurses, psychologists), who employ validated screening tools and evidence-based psychotherapeutic interventions to assess and manage outpatients [25]. As shown by the present study, inpatient CL services can be an area for applications of the collaborative care framework, particularly via a collaborative care team consisting of psychiatrists and the psychology extern working as consultants in the inpatient medical setting for patients overseen by a primary medical team.

There is extensive evidence for the impact of collaborative care models in managing psychiatric conditions. For instance, the Improving Mood-Promoting Access to Collaborative Treatment (IMPACT) Trial showed a 50% improvement in outcomes for depression treatment in the collaborative care intervention compared to the standard of care group [26]. Subsequent studies have shown the efficacy of collaborative care in treating depression and anxiety in outpatient settings [27]. Collaborative care models may also improve treatment adherence, quality of care, and clinical outcomes for patients with mental illness and chronic medical conditions, such as those with both depression and diabetes [28]. Given that collaborative care has illustrated efficacy in mental healthcare delivery, the time is ripe for integrated psychiatry-psychology teams to be more widely adopted in CL psychiatry services.

Lastly, the collaboration of psychiatrists with a psychology extern provides a valuable educational opportunity. It is critical that psychologists and psychiatrists view one another as essential components of the team. A qualitative study on the perspectives of team members (e.g., nurses, psychiatrists) on the role of psychologists in inpatient psychiatric units found that while psychologists were perceived as integral to the team, many members needed a clearer understanding of their roles [29]. Therefore, integrating trainees on the same team (e.g., psychology externs with resident psychiatrists) may facilitate the training of clinicians to be more comfortable in interdisciplinary teams and promote mutual understanding of roles from the start of their careers [9]. Psychiatry-psychology collaboration thus serves as a valuable opportunity to enhance teamwork, communication, and education for providers.

The inferences drawn from the present study are limited by the presence of a single academic site and one psychology extern, limiting the generalizability of study interpretations. However, the purpose is to describe the experiences of the psychology extern and provide examples of how collaborative care has been implemented in the CL setting at a particular site. By bringing attention to the need and potential benefits of this collaborative model, we aim to promote broader awareness, implementation, and investigation of integrated psychology-psychiatry collaboration across other inpatient CL programs.

Recommendations for integrated psychiatry-psychology collaboration in CL services

**Figure 1:**
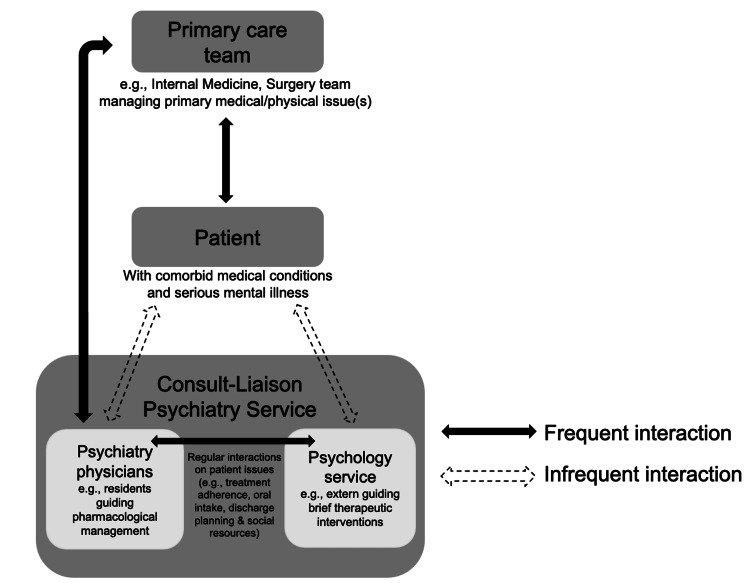
Application of a Collaborative Care Framework for CL Psychiatry Services The CL psychiatry team should consist of multidisciplinary behavioral health providers, including training psychiatrists and a psychology extern. The extern frequently engages in team rounds and care decisions, while providing individualized therapeutic interventions that can aid in patients’ coping with illness and hospitalization.

Given the presence of maladaptive coping among patients with SMI, the versatile roles of a psychology extern described in the present study, and the efficacy of collaborative care, we therefore propose the following recommendations for leveraging a collaborative care framework to integrate psychology externs into CL psychiatry services:

1. The inpatient collaborative care team should consist of psychiatrists from the CL team and psychology services from the CL team. Figure 1 demonstrates a proposed model of collaborative care applied to the CL psychiatry service as a consultant team in the inpatient setting. As shown in the present study, the potential benefits of this collaborative structure include improved patient coping behaviors and the ability to engage in treatment and care planning.

2. The CL service should utilize the fully integrated level of collaborative care to provide psychological services in the inpatient medical setting. In the present study, the psychiatry-psychology collaboration employed by the CL service approaches levels 5-6 of collaboration and exhibits elements of integrated collaborative care, including frequent in-person communication, formal and informal team meetings, and a shared belief for team-oriented care (Table 3).

3. All members should have a clear understanding of the culture and everyone’s roles and responsibilities. In the present study, the role of psychiatrists included prioritizing medication concerns and liaising with the primary medical team, while the psychology extern complemented these responsibilities by spending more time with patients during therapy sessions to enhance coping behaviors. To facilitate an understanding of roles, responsibilities should be delineated throughout a patient's course of care and understood and accepted by both the psychiatrists and psychology extern, with a shared goal to improve patient outcomes. Opportunities to strengthen a culture of learning and communication between interdisciplinary team members should also be facilitated, such as during patient rounds, with interprofessional team members encouraged to engage in timely, consistent, and transparent communication with a shared awareness of patient goals [30].

4. Brief psychotherapeutic interventions should be provided to adult inpatients with the goal of improving coping skills and behaviors that can aid in the management of medical illness and course of hospitalization (e.g., medication adherence, interactions with healthcare staff). These interventions should be individualized and selected according to patients’ diagnoses and unique coping mechanisms, and offered by a trained professional with awareness of evidence-based techniques effective for an acute inpatient setting.

5. Outcome measures such as systemic factors (e.g., hospital readmission rates), patient-reported symptoms (e.g., depression symptoms), and provider-reported perspectives (e.g., perceptions of an interdisciplinary provider team) should be assessed via subsequent studies to evaluate the efficacy of brief therapeutic interventions and quality of care delivered.

## Conclusions

By examining the experiences of a psychology extern through the lens of a collaborative care framework, we highlight the need for broader implementation of an integrated model of psychiatry-psychology collaboration in CL psychiatry teams. The care of patients hospitalized with comorbid medical conditions and SMI is complex, given the significant psychosocial stressors and emotional dysregulation often faced by these populations. Leveraging the expertise of a psychology extern in CL psychiatry teams can enhance patients’ coping skills and inpatient experiences. Lastly, recommendations for utilizing a collaborative care framework were presented to better facilitate the integration of psychology externs into CL psychiatry teams. Future studies are needed to investigate outcomes of psychiatry-psychology collaboration on CL services, particularly regarding patients’ quality of life, clinical outcomes, and provider perspectives.
